# The Pathology and Physiology of Diabetes

**Published:** 1887-11

**Authors:** 


### The Pathology and Physiology of Diabetes. By Prosper Bender, M. D., of Boston. 1887.

This pamphlet is the printed essay read before the Massachusetts Homœopathic Medical Society in April last.

It is an interesting, clear, and able review of all the researches and theories, down to the present time, of all the great authorities who are endeavoring to account for the presence of sugar in the urine in the well-known disease, diabetes.

It seems very probable that as the physiology and the pathology of the brain are more and more developed, we will see that many diseases are caused by neurotic lesions. Among the most important of these diseases will be found those of the liver and kidneys. So it seems to us that Dr. Bender is in the right in considering diabetes a neurosis. In this essay on diabetes many authorities are quoted and much interesting matter adduced.

